# Role of Paper-Based Sensors in Fight against Cancer for the Developing World

**DOI:** 10.3390/bios12090737

**Published:** 2022-09-07

**Authors:** Amey Dukle, Arputharaj Joseph Nathanael, Balaji Panchapakesan, Tae-Hwan Oh

**Affiliations:** 1 Centre for Biomaterials, Cellular and Molecular Theranostics (CBCMT), Vellore Institute of Technology (VIT), Vellore 632014, Tamil Nadu, India; 2 Small Systems Laboratory, Department of Mechanical Engineering, Worcester Polytechnic Institute, Worcester, MA 01609, USA; 3 School of Chemical Engineering, Yeungnam University, Gyeongsan 38541, Korea

**Keywords:** paper-based sensors, cancer screening, disposable sensors, sensors, paper fluidics, microfluidics

## Abstract

Cancer is one of the major killers across the globe. According to the WHO, more than 10 million people succumbed to cancer in the year 2020 alone. The early detection of cancer is key to reducing the mortality rate. In low- and medium-income countries, the screening facilities are limited due to a scarcity of resources and equipment. Paper-based microfluidics provide a platform for a low-cost, biodegradable micro-total analysis system (µTAS) that can be used for the detection of critical biomarkers for cancer screening. This work aims to review and provide a perspective on various available paper-based methods for cancer screening. The work includes an overview of paper-based sensors, the analytes that can be detected and the detection, and readout methods used.

## 1. Introduction

Cancer is a major cause of death worldwide [1,2]. It is estimated to be the cause of every 1 in 6 deaths [3,4]. According to the World Health Organisation (WHO), in the year 2020 more than 10 million people lost their life to cancer [5]. Worldwide, an estimated 19.3 million new cancer cases and almost 10.0 million cancer deaths occurred in 2020 [6]. Although the causes of cancer may vary depending on the type, it has been observed that the incidence rate of disease is on the rise [7,8].Worldwide, by 2040, 28.7 million new cases of cancer are projected to occur, a 47% rise compared to 19.3 million in 2020. An increase in the global cancer burden in the next fifty years will come from low- and middle-income countries (400% in low-income countries, 168% in middle-income countries, and 53% in high-income countries) [9,10]. Though there are different treatment strategies that have been developed and the disease is no longer ’incurable’ [11,12,13,14], the success rate of the treatment depends on the stage of disease progression [15,16,17]. An individual undergoing treatment in the early stages of cancer has a many times higher chance of survival than in the later stages [18,19]. Estimates suggest that approximately 30–50% of cancer deaths can be prevented by early detection and treatment [20,21,22].

Although the incidence rate of cancer is higher in wealthy nations compared to low- and middle-income countries, low- and middle-income countries have a lower survival rate, partly due to the late presentation of the disease [23]. The barriers of cancer care in developing countries are due to late-stage presentation, quality of care, affordability, and a lack of access to advanced clinical resources. Late-stage presentation puts tremendous burden on clinicians [10]. The premature death and loss of productive life in the working population results in a significant economic impact on these countries.

In low- and middle-income countries, access to health care facilities is not readily available [24,25,26,27,28]. The number of physicians in low- to middle-income countries can be as low as 0.1 to 2 per 1000 people. In many cases, they are heavily burdened, leading to long waiting times [29]. In many low- and middle-income countries, cancer screening is not covered under insurance, thus discouraging patients from undergoing cancer screening.

To address the issue of cancer screening in these countries, various strategies have been developed [30]. These screening devices need to be easy to manufacture, low-cost, portable, and should not require any special training. Paper-based sensors are gaining significance in this field as they possess all the features of an ideal screening device [31,32,33,34,35,36,37,38,39,40,41,42,43,44,45,46,47,48,49,50].

Paper was invented by the Egyptians in the fourth century BC. It is one material that has existed continuously since the beginning of Egyptian civilization. Paper-based products are the most sold products in the world. For example, the Bible is the most sold book in the world and is printed on paper. The question then becomes: can we print cancer screening devices on paper to enable their use as low-cost sensors for cancer screening? Paper-based sensors for cancer screening are akin to an at-home pregnancy test kit. These kits will indicate whether a specific cancer biomarker is present in the person’s body. In many cases, the result will be qualitative, i.e., yes/no type. However, novel sensors have been developed that provide a quantitative output [51].

In case the test returns a positive result, the patient can consult medical professionals so that exhaustive testing can be performed and the stage of the disease identified.

The most common sensing principle used in paper-based sensors for cancer screening is ELISA, wherein the analyte is labelled and detected using a sandwich assay [52]. The results are then readout using a plate reader to specify analyte concentration against a standard curve [53,54,55,56,57]. Various other methods are also being explored for the development of sensors.

This work reviews the various paper-based sensors that have been developed for cancer screening. In the next sections, a brief introduction about paper-based sensors, the analytes used for cancer screening, and the detection and read-out techniques used are discussed.

## 2. Paper-based Sensors: Low-Cost Screening Devices for the Developing World

Advancements in microfluidics have led to their widespread usage in sensing applications. Microfluidics, as the name suggests, uses small fluidic channels where liquids, proteins and cells can be manipulated through flow control [58]. The channels are designed in such a way that various operations such as separation of phases, biological cells, the size-based separation of particles, to name a few, can be performed. However, fabrication of microfluidic devices may not be possible in low-income countries due to the lack of availability of materials. Even poly dimethyl siloxance (PDMS)-based soft lithography techniques require a master that can be expensive to make in low-income countries and set-ups such as mask makers and lithography may not be readily available. Paper is a low-cost alternative for sensors in the fight against cancer that may not need lithographic processing. Droplet-based paper devices could be highly useful for biomarker detection. Even microfluidic devices using screen-printing techniques can be printed on paper.

Paper-based sensors are devices that are fabricated on a paper substrate. These devices are printed on a cellulose-based paper substrate using readily available printers, making them easily accessible.

A typical paper-based sensor is based on exploiting the capability of paper to wick liquid, leading to capillary action in the paper. A typical paper-based sensor is developed on an hydrophilic paper substrate with wicking capabilities. Using surface treatment methods, a hydrophobic barrier is created in order to guide the flow of liquid through the specific path or channels.

The most commonly used paper in the fabrication of paper-based sensors is filter paper. Due to its pores, it has sufficient wicking capabilities, providing a moderate flow rate. Whatmann-branded filter paper manufactured by General Electric Health Care is the most widely used filter paper due to its uniform pore size and distribution. In applications where filter paper is not suitable, nitro-cellulose paper is used as a substrate. The main advantage of nitrocellulose substrate is its easy and efficient binding of proteins [59].

For example, nitrocellulose film is used for protein immobilization and filter paper is used for its water absorption. In one particular study for detection of bladder cancer, a glass-cellulose film was used for sampling, a nitro-cellulose film was used for protein immobilization, and filter paper was used for sample transfer due to its adsorption capabilities [60].

Table 1 highlights the different types of paper substrates that have been developed for paper-based sensing applications.

**Figure 1:**
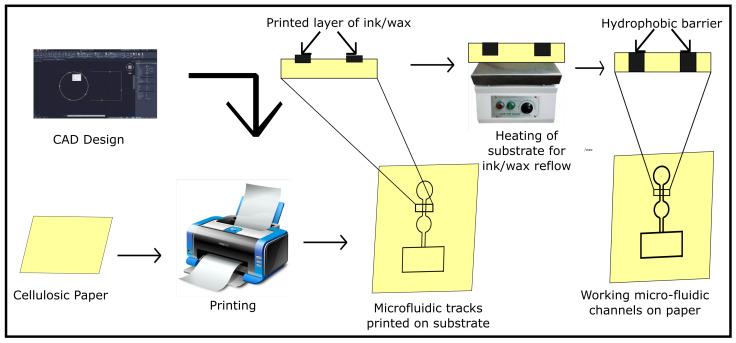
Workflow of the fabrication process for printing microfluidic channels on a paper substrate.

For patterning of the microfluidic channels on paper substrate, various printing techniques such as wax-printing, ink-jet printing, screen printing, lithography, plasma processing, and manual pattern drawing have been explored. In a typical printing-based fabrication process, a CAD model of the microfluidic channels is printed using wax/ink-jet–printer on the substrate. Since the model is printed on only one side of the substrate, the substrate is heated to cause the reflow of the hydrophobic ink/wax barrier over the complete cross-section of the substrate. These barriers act as microfluidic channels guiding the flow of the fluid (Figure 1).

Although paper-based microfluidic channels can be used for most of applications as conventional microfluidic chips, there may be concerns regarding the utility of paper substrate for use in microfluidic applications requiring multiple layers of fluidic channels for phase separation applications. For such applications Japanese paper, folding techniques such as origami and Kirigami are being explored [87].

The low cost of substrate, the ability to print continuously, the ability to make arrays of devices, and minimal capital requirements for the fabrication setup make paper-based sensors an interesting candidate for use in mass screening process. Paper being a naturally derived substance is biodegradable in nature. This will also reduce the environmental impact due mass production and usage of screening kits.

## 3. Design and Working of a Typical Paper-Based Sensor

The paper-based sensor used for screening must be fast, accurate, reliable, and must have a low limit of detection. A low limit of detection will allow for successful detection even during the early onset of cancer. A typical sensing setup comprises the following elements:

- Analyte: It can be simply be defined as the chemical substance to be measured. In the case of cancer screening, cancer specific biomarkers, tumour markers, antigen, and proteins are essential analytes. More about the different types of analytes for cancer screening in Section 4
- Labeling: In most of the biosensors, labeling plays an important role. For the detection of the analyte, labels that attach to the molecule are used. The selection of label depends on the detection method used.
- Recognition: The recognition element is used to convert the biological information into signals. The most common detection method used in cancer screeing is enzyme-linked immunoassay (ELISA). In Section 5, various recognition methods that have been used for paper-based cancer screening are discussed.
- Readout: The readout method is used to obtain the outcome of the test. Some common readout methods are electrochemical, optical, and colorimetric. Depending on the detection technique used, the results obtained can either be qualitative (yes/no) type or quantitative (numerical values).

**Figure 2:**
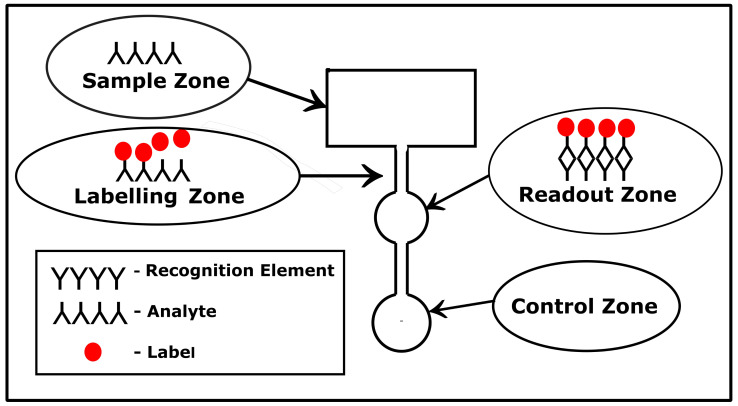
Typical layout of a paper-based sensor with the different zones. Each zone performs a specific function.

The paper-based microfluidic platform is divided into different zones, with each zone having a specific functionality, as shown in Figure 2. In the sampling zone, the sample is placed on the paper substrate. While passing through the microfluidic channels, labeling elements get attached to the analyte. In the detection zone, the analyte is detected and a signal is generated for the readout. In the case of colorimetry-based devices, there is a special zone termed as ’control zone’. In case a test yields a positive or negative result, there is a color change observed in the control zone.

## 4. Analytes for Cancer Screening

For cancer detection, biomarkers play an important role. According to the National Cancer Institute (NCI), biomarkers are defined as “a biological molecule found in blood, other body fluids, or tissues that is a sign of a normal or abnormal process, or of a condition or disease” [88]. Though biomarkers may be generated due to various factors such as somatic mutations, transcriptional changes, or post-translational modifications, they an important differentiators for an affected individual compared to a healthy individual [88]. A handful of biomarkers have been approved for cancer detection. For example, high levels of carcino embryogenic antigen may mean the presence of cancer. Similarly, CA 125 is a protein that is detected in blood for ovarian cancer. Lysophosphatidic acid, leptin, osteopotin, and insulin-like growth factor receptor 2 are used as a biomarker for ovarian carcinoma. Early prostate cancer antigen 2 is used as a novel biomarker for prostate cancer [89]. Biomarkers are not only useful for the detection of screening of cancer, they are also used in monitoring the effectiveness on any treatment or therapy. Various biomarkers are present in blood, saliva, urine, stools, etc., making it possible to obtain samples for analysis in a non-invasive or minimally invasive manner. There are various biomarkers that are used for cancer screening, including antigens, micro-RNAs, proteins, antibodies, and tumor cells. For example, the antibody–antigen interaction creates a signal that is measured qualitatively or quantitatively on a paper substrate.

## 5. Recognition Element

Recognition elements are responsible for the recognition of target analytes (ex: receptors) and their conversion into a signal, which can be qualitative, semi-quantitative, or quantitative [90]. An ideal recognition element has a highly specific binding affinity towards the analyte of interest [90]. Recognition elements can either be natural or artificial bio-molecules that are synthetically obtained [90,91,92,93,94]. When the target analyte molecule attaches to the recognition element, it undergoes a biochemical reaction producing a signal [90]. In some cases, for the detection of analyte, labeling agents such as nanoparticles are attached to the analyte molecule [90].

### 5.1. Antibodies

Antibodies are a type of natural bio-receptor that can be derived from living organisms [90]. Monoclonal antibodies are widely preferred in cancer screening applications due to their high specificity to the target antigen [95,96]. Through the development of hybridoma technology, it is possible to obtain a reliable and uniform supply of monoclonal antibodies [96]. This has also led to better reliability and accuracy of sensors using monoclonal antibodies. Covalent binding of antibodies to cellulose paper discs has been developed for colorimetric immunoassays. The antibodies were coated on the amine-functionalized cellulose paper discs. Through a glutaraldehyde cross-linking agent, the antibodies showed enhanced binding activity to the target when compared to the periodate oxidation method [97]. Other methods for antibody immobilization on a paper with shelf-life up to 12 months have been described [98,99]. Polyclonal antibodies have multiple binding sites, each specific to a particular antigen. Although they are cheaper to produce compared to monoclonal antibodies, they are not suitable for sensing applications due to the multiple binding sites [100].

For the detection of analytes, the principle of enzyme linked immunosorbent assay (ELISA) is applied in sensor design. Nanoparticles such as gold are used as antibody carriers and signal enhancers for ELISA. In the range between 0 and 60 U/mL, the ELISA assay adopting gold nanoparticles as an optical signal enhancer resulted in higher sensitivity and shorter assay time when compared to classical ELISA procedures. This was used to detect breast cancer biomarkers [101].

A sensor for the detection of prostate-specific antigens (PSA) used multiwalled carbon nanotubes (MWCNT) activated with anti-PSA antibody for the detection [102]. Due to the site-selective interaction between the antigen and the antibody there was a change in the resistance. This change in resistance could be measured using a benchtop multi-meter [102]. This method was found to be cheaper and faster than the ELISA method used in cancer diagnosis [102]. Similarly, single-wall carbon nanotube based biosensors have been used in the identification of cancer antibody–antigen interactions in blood samples using electrical conductance measurements. Following the measurements, a classification algorithm was implemented to differentiate between cancer and controls with 90% accuracy [103,104,105].

**Figure 3:**
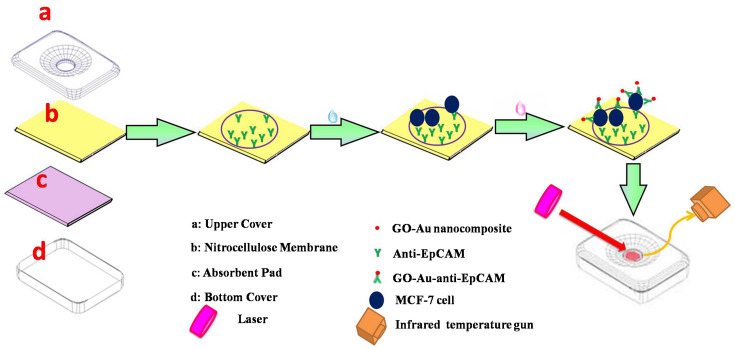
Working of photothermal effect based sensor for MCF-7 cancer cell detection. (Reproduced with permission from [107] Copyright 2016, Elsevier).

In a colorimetric sensor for the detection of pancreatic cancer biomarker (PEAK1), gold nanoparticles that were used as a labeling element acted as a color dye catalyst to produce colorimetric signals [106]. A photothermal-effect-based sensor used a graphene oxide (GO)-gold–anti-EpCAM antibody composite as the recognition element for the detection of MCF-7 cancer cells specific antigen [107]. After laser irradiation at the test zone, the temperature contrast was recorded for the detection of cell numbers (Figure 3) [107].

### 5.2. Aptamers

Aptamers are single-stranded nucleic acids that are folded into a specific architecture [108,109]. Due to their specific binding of the target proteins, they are used for sensing applications [108,110]. Their size and chemical stability make them widely preferred for the detection of proteins and small molecules [110,111]. Their low cost makes them a preferred alternative to antibodies in sensing applications [111]. For example, carbon-nanotube-based RNA apatamer sensors were developed for detecting IL6 in blood samples. Apatamer sensors based on field effect transistor arrays suggested a shift in drain current versus gate voltage for 1 pg and 1 ng of IL-6 exposure. The concentration of 1 pg falls below the diagnostic gray zone for cancer (2.3 pg–4 ng/mL), which is an indicator of early-stage cancer [112].

For the design of aptamer-based sensors, various strategies such as sandwich, target-induced structure switching, or competitive replacement modes have been used for biosensor design [111]. Electrochemical sensing is the most preferred sensing method with aptamer as the recognition element; however, other methods such as optical sensing have also been explored [110].

In a fluorescence-based paper-based sensor designed for the detection of multiple types of cancer cells, graphene oxide-coated with mesoporus-silica-labelled high-specificity aptamers was used as a labeling element [113]. Using the excitation wavelength of 350 nm, a color change was produced that could be observed through naked eye [113].

## 6. Sensing and Readout Methods

For achieving higher utility of paper-based sensors, the readout method used should be cost-effective and portable. The method provides fast and accurate results without a requirement for extensive handling by experts.

In most of the cases, qualitative readout methods should suffice. However, with focus on providing health professionals with important data at the point-of-care, qualitative readout methods are also gaining significance. Although there are various readout methods for sensing applications, electrochemical and optical are the most widely used readout methods for paper-based sensing applications. With advancements in smartphone and machine learning technologies, there have been works that use smartphones for signal interpretation and readout.

### 6.1. Modified Electrodes

For electrochemical sensing, the potential difference between the electrodes is proportional to the concentration of the analyte. In paper-based sensors, the working electrode is modified such that the binding of the analyte produces an electrical signal through a change in resistance, current, capacitance, or impedance.

**Figure 4:**
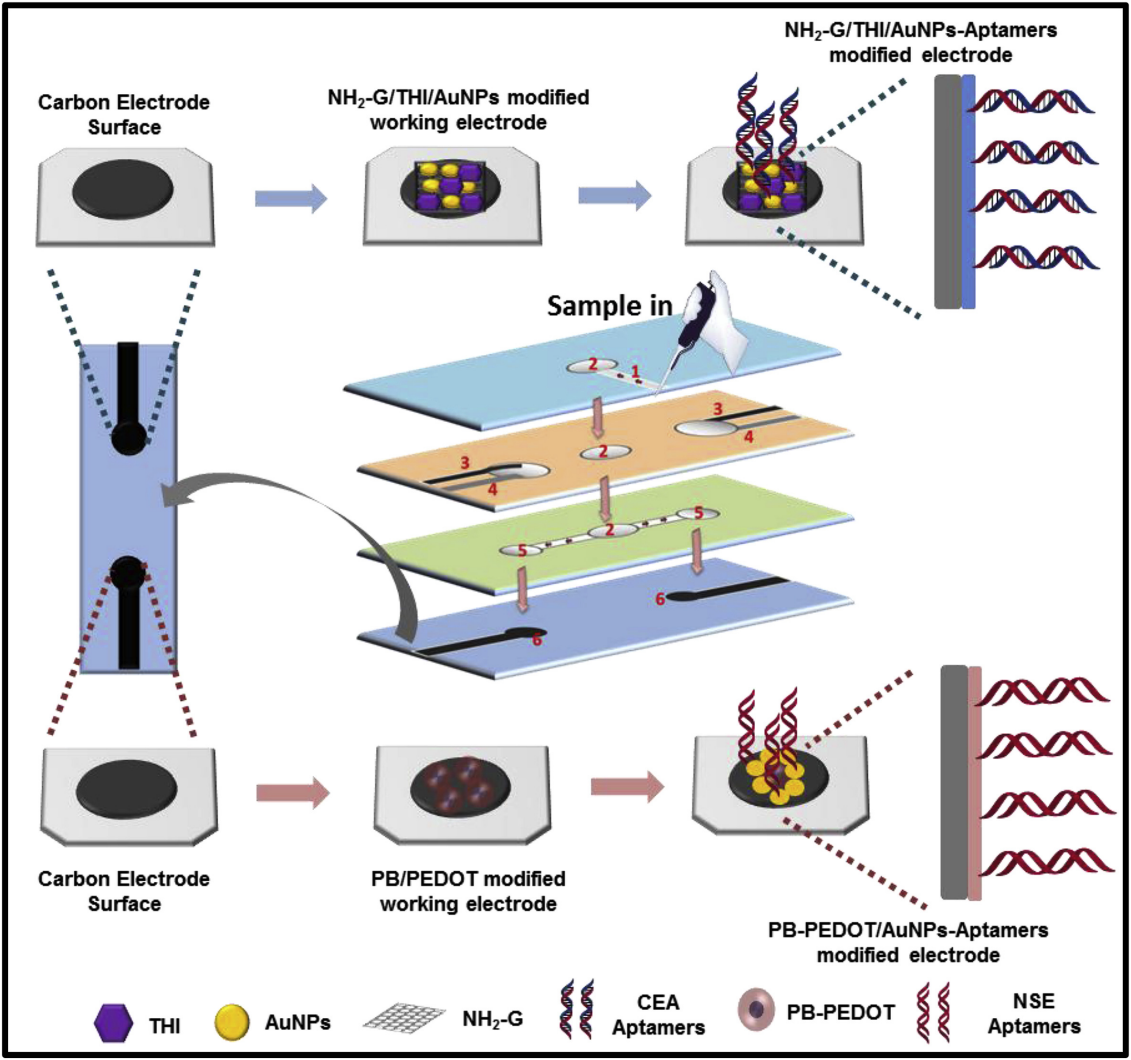
Mechanics of modified electrode paper-based apta sensor. (Reproduced with permission from [116] Copyright 2019, Elsevier).

Various nanocomposites have been used for the fabrication of modified electrodes. These nanocomposites perform a dual function: recognition and amplification. Amino functional graphene (NH2-G)/thionine (Thi)/gold nanoparticles (AuNPs) nanocomposites are coated with recognition elements such as üimmobilized anti-CEA [114] and anti-NSE [115] for the detection of specific analytes. The sensor could provide fast results with a low limit of detection of 10 pg/mL [114]. In a more recent work, an aptasensor with two working electrodes capable of the simultaneous detection of CEA and NSE has been developed. Along with the NH2-G/Thi/Au nanocomposite, Prussian blue (PB)- poly (3,4- ethylenedioxythiophene) (PEDOT)- AuNPs nanocomposite was used for the fabrication of the second electrode, which was coated with immobilized CEA and NSE aptamers [116]. The device worked on the principle of electrochemluminescnce and could achieve fast and accurate detection of CEA and NSE with a limit of detection of 2 pg/mL and 10 pg/mL, respectively (Figure 4) [116].

### 6.2. Electrochemical

In electrochemical sensing method, the analyte generates an electrical signal proportional to its concentration [90]. The signals may be generated through a biorecognition event, modified electrodes, or enzyme mediated electrodes [90,117]. For electrochemical detection, the sensor should have three electrode systems with reference, working, and counter electrodes. For measuring the signals, electrochemical devices such as electrochemical workstations or bench-top multimeters are used.

Various routes such as the use of labeling agents or modified electrodes may be used for generating electrical output from biological signals. In a sensor developed for the detection of cancer antigens, a marker for ovarian cancer, a reduced graphene oxide/gold nanoparticle/thionine nanocomposite was used as working electrode [118].

For the reliable detection of signals, signal amplification techniques are used. In paper-based sensors for the detection of CEA using horseradish peroxidase (HRP)–O-phenylenediamine–H${}_{2}$O${}_{2}$ as a detection element, graphene was coated on the substrate for accelerating the electron transfer and amplifying the signals [119].

### 6.3. Optical

For optical sensing, signals are generated through a recognition process by the formation of an antigen–antibody complex [90]. The optical signals could be fluorosence, chemiluminescence, or color change [90]. Other than the signals that display a direct color change, a photo-detector is used for measuring the signals [90].

**Figure 5:**
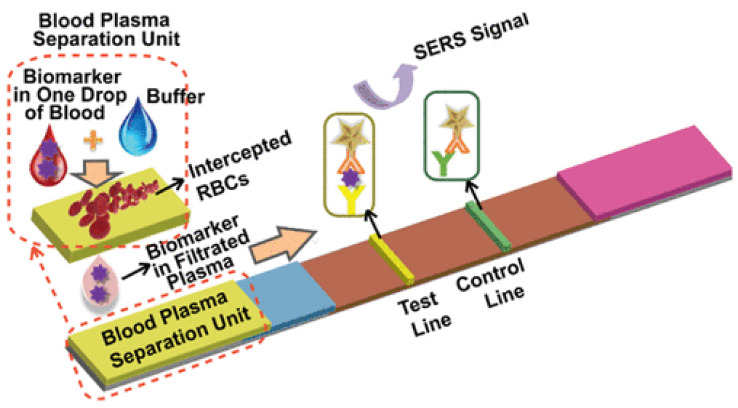
Working of a paper-based sensor for CEA concentration detection using surface-enhanced Raman scattering (SERS). (Reproduced with permission from [120] Copyright 2021, American Chemical Society).

Surface-enhanced Raman scattering is a popularly used method for signal detection in paper-based sensors. Gold nanostar@Raman reporter@silica-sandwiched nanoparticles have been developed as surface-enhanced Raman scattering (SERS) probes for the paper-based lateral flow strip (PLFS) assay [120]. A sensor for the detection of CEA used a portable raman sensor for measurement (Figure 5) [120]. Using a paper-based lateral flow strip capable of plasma separation and using silica nanoparticles for labeling the sensor displayed a limit of detection of 1 ng/mL [120].

For naked eye detection, luminiscent reporters are used as labeling elements These can be nanoparticles [51], conjugated polyelectrolytes [121], or multi walled carbon nanotubes [102].

### 6.4. Smartphone/Machine-Learning-Based

Smartphones are devices that are readily available, even in low- and middle-income countries. Mobile health is becoming increasingly popular in developing countries [122]. It is widely explored as a tool for the efficient delivery of services, including in healthcare. Smartphones have been explored as a readout method for both optical and electrical signals [123,124,125]. For optical signals, a smartphone camera is used for data acquisition [126,127,128]. Using a custom application, the acquired image is compared with reference values and the result is calculated [126,127,128,129,130].

Smart phone-based imaging was used for calculating and displaying results in a multi-layered paper-based sensor for cancer screening [131]. The movable layers allowed one to control the flow of the solution. Using the special design and smartphone-based readout, it was possible to achieve a low detection limit of 0.015 ng/mL [131].

**Figure 6:**
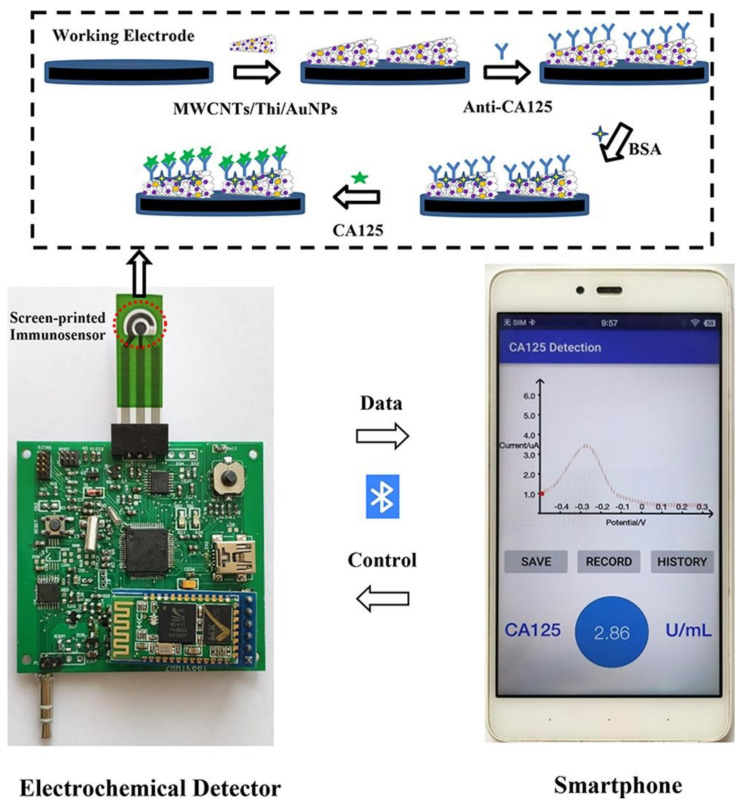
Sensor for detection of cancer antigen (CA125). The screen-printed sensor used electrochemical deterrence for signal measurement and a smartphone with a custom application for readout (reproduced with permission from [132] Copyright 2022, Elsevier).

Smartphones are also used for coupling with an electrochemical sensing device for the readout of signals [132]. A screen-printed sensor with multi-walled carbon nanotubes (MwCNT)/thionine (Thi)/gold nanoparticles (AuNPs) electrodes is capable of detecting cancer antigen (CA125) with a limit of detection of 2mU/mL [132]. The sensor uses a electrochemical detector powered using a smartphone, and it transfers data to the smartphone, where it is readout using a custom app (Figure 6) [132].

Table 2 summarizes recent works using paper-based sensors for cancer screening. It provides the breakdown of the sensor in terms of the biomarker(s) detected, recognition element, readout method used, and the types of cancer detected.

## 7. Limitations of Current Paper-based Methods

Despite the promising future, there are currently several limitations that hinder the large-scale acceptance of paper-based sensors. Ranging from fabrication methods, a requirement of measurement equipment, to regulatory requirements, many limitations need to be addressed before paper sensors are actually put into service. Sensitivity and accuracy are also concerns in paper-based devices.

However, paper-based sensors are excellent for qualitative and semi-quantitative screening. One can further improve the accuracy of paper-based testing through implementing paper-based testing with artificial-intelligence-based analysis. Machine learning and deep learning can even predict the sequence of DNA and RNA that can point to cancer mutations. They could also potentially detect cancer cells in blood droplets. The ability to differentiate between cancer versus normal cells in blood based on AI can be a very powerful approach in making paper-based testing a reality for mass screening [152].

With the exception of laser printing and screen printing, different fabrication methods used for the fabrication of paper-based microfluidic devices such as photo-lithography, e-beam lithography, reactive ion etching, and metal or oxide deposition require extensive capital investment and training, making them non-feasible for use in low- and middle-income countries.

For devices with qualitative measurement of signals, despite the low cost of single paper-based sensing device, expensive equipment such as electrochemical workstations, photo detectors, or electrochemiluminscence detectors are required for a results readout. This not only affects the portability of the device but also increases the overall cost of the screening, and it requires a trained technician to carry out the readout.

Nevertheless, paper-based sensors can make a significant impact in terms of testing blood for infectious diseases and even in the fight against cancer. The qualitative assessment of whether a person has cancer based on biomarkers in blood can reduce the cancer clinical burden in low- and middle-income countries. The paper-based testing method will definitely have an advantage here to reduce the clinical burden through the large-scale screening of populations. People with cancer undergoing chemotherapy may be prone to infectious diseases. Life-threatening infectious diseases can kill people in a few days. Here, POC systems based on paper and colorimetric detection methods for parasites, viruses, and other agents would be highly valuable. Before vaccination, people, including doctors, were dying of COVID-19 within a few days to two weeks. Low-cost qualitative paper-based sensors with instant results through color change are an absolute necessity to differentiate between population who has the virus from those who do not. This may be cheaper than the current PCR test that is used and is done in the laboratory. In the era of the pandemic, low-cost sensors for personal safety are very important. Qualitative paper-based sensors with instant read out will be highly useful in such pandemics as one cannot run tests in the laboratory frequently.

## 8. Conclusions

Paper-based sensors have immense potential to to act as low-cost tools in the mass screening of cancer. They can be used in cancer-screening camps, especially in low- and middle-income countries. Due to the health care resources being highly stressed in these countries, screening using paper-based sensors will act as a filter. The samples of the patients who test positive in the screening stages can then be treated as a priority. This will in a way help in resource allocation and management in these countries. It must be mentioned that the paper-based sensors with their current state of the art are insufficient for providing data to healthcare professionals in making important decisions. Further, testing will be required to ascertain the stage of the disease before any treatment plan is decided. Many times, the same biomarker is produced for multiple types of cancers. CEA is a common marker for multiple types of cancer such as lung, breast, ovaries, stomach, and intestine, to name a few. Thus, it is necessary to determine the type of cancer and the stage of disease progression before starting the treatment. Normal CEA levels are 2.5 ng/mL. A CEA level of 10 ng/mL would indicate the presence of cancer, and anything above 20 ng/mL would indicate the spread of cancer.

Patients with continuously decreasing levels of CEA do better after treatment than patients with increasing CEA levels. A regular paper-based CEA test with an instant read out can tell the doctors the potential for cancer progression. Their use will reduce the number of expensive tests (CT scans, PET-scans, etc.) a patient undergoes during follow-up visits and also help reduce the cost of treatment. In the era of telemedicine, it is becoming increasingly convenient to deliver healthcare at home. Cancer-screening tests can be conducted at home, and the results of the test can be emailed or texted to a doctor automatically using smart phones. So, paper-based testing is important here for home-based low-cost sensors.

Although paper-based sensors may not potentially be the knight in shining armor against humanity’s fight against cancer, they can potentially be the important foot soldier in the fight. Future integrated paper-based sensors where all the sensing and electronic circuitry are printed in paper can make an impact on low-cost testing. Paper-based cantilevers with optical sensors and electronics integrated in a hand-held chip could enable the detection of cancer biomarkers such as prostate-specific antigen (PSA) from blood samples. Thus, there are exciting opportunities for paper-based sensors in the fight against cancer. The places where paper-based sensors along with mobile phones and AI-based techniques could make an impact in the fight against cancer are (1) the low-cost qualitative screening of large populations; (2) reducing the clinical burden through proper resource allocation; (3) estimating cancer prognosis; (4) monitoring cancer treatment; and (5) detecting cancer recurrence qualitatively or semi-quantitatively. With the ability to miniaturize anything, from detectors to spectrometers, one can implement miniaturized low-cost detectors along with paper-based sensors for cancer detection. Such miniaturized sensors are already made by many companies and could be bought off the shelf and integrated with a paper-based sensor. Finally, paper-based methods with artificial intelligence techniques can enable low cost and further improve the sensitivity and accuracy of paper-based sensors.

## Figures and Tables

**Table 1:** Paper substrates of interest for paper-based microfluidics.

Paper Type	Properties	Sensing Methods	Applications/Notes/References
Whatman Filter Paper Grade 1	Size: 26 × 31 mm to 600 × 600 mm sheets or 10 mm to 150 cm circles. Porosity: 11 µm Nominal thickness: 180 µm Medium retention and flow rate	Colorimetric, Surface Plasmon Resonance SERS, Electrochemical, Chemiluminescence, Phosphorescence, Photometric, Chromogenic sensing, Fluorescence, Dye based sensing, Spectrometry	Analytical separation [61] Electrophoretic separation [62] Soil analysis [63,64,65] Food testing [66] Point of care testing [67] Protein [68] Atmospheric dust [69] Gas detection [70] HIV detection [71] Explosive Sensing [72] Automated DNA extraction and amplification [73]
Whatman Filter Paper Grade 2	Size: 460 × 570 mm to 580 × 680 mm or 42.5 mm to 500 cm circles. Porosity: 8 µm Nominal thickness: 190 µm More retention than Grade 1 and slower flow rate ** **	Same sensing methods are applicable as in Grade 1	Same applications as Grade 1 except slower flow rate and higher retention due to smaller pore size.
Whatman Filter Paper Grade 3	Size: 26 × 31 mm to 600 × 600 mm or 23 mm to 320 mm circles. Porosity: 6 µm Nominal thickness: 390 µm More retention than Grade 1, 2 and slower flow rate	Poor colorimetric sensing due to slower flow rates	Same applications as Grade 1 except slower flow rate. Poor for colorimetric sensing due to lower color contrast
Whatman Filter Paper Grade 4, 5, 6	Main difference is porosity; Grade 4: 25 µm Grade 5: 2.5 µm and Grade 6: 3 µm.	Poor colorimetric sensing of Grade 5 and 6 is expected due to slower flow rates.	Same applications as Grade 1 except slower flow rate of Grade 5 and 6. Grade 4 suitable for large particles monitoring in air. Soil Suction Testing [74]
Whatman® Grade 903	W × L = 450 mm × 450 mm, 140 µm thickness, porosity: 4–7 µm	Compatible with most sensing methods. Super refined cellulose	Whole-blood collection [75], HIV load, and drug-resistance testing [76]. Element detection in neonatal blood spots (NBSs) using sector-field inductively coupled plasma-mass spectrometry [77].
Whatman® FTA filter paper cards	N/A	Highly sensitive for rapid nucleic acid extractions and storage.	Nucleic acid extraction from cells [78]; fine needle aspirates for cancer testing [79]; tissue analysis [80]; and virus and bacterial RNA detection and preservation [81].
Nitrocellolose membrane	pore size: 0.2 µm	Same sensing methods are applicable as in Grade 1	Western Blotting [82] Fabrication of Lateral Flow Assay [83]
Nanocellulose paper	Nanofibrillated cellulose (NFC) coated with layer of reactive nanoporous silicone nanofilament	Mainly restricted to applications requiring hydrophobic substrate	Paper-based electronics [84]
Microcrystaline Cellulose/ Polyvinyl Alcohol Paper	Porosity: 90%, pore size (between 23 and 46 µm), thickness (from 315 to 436 µm), and high light transmission under water (>95%)	Similar to nanocellulose paper	low-cost cell culture platform [85]
Omniphobic RF paper	“fluoroalkylated paper” (“RF paper”) by vapor-phase silanization of paper with fluoroalkyl trichlorosilanes	Resist wetting by liquids with a wide range of surface tensions correlates with the length and degree of fluorination of the organosilane and with the roughness of the paper	Same as nanocellulose paper [48]
Photo paper	Commercially sold by Epson, Canon etc.	Same applications as Grade 1	Pumpless paper-based analytical devices [86]

**Table 2:** Paper-based sensors for cancer screening.

Biomarker Detected	Recognition Element	Readout Method	Types of Cancer	Reference
MCF-7 Cells	Graphene Oxide- Gold nanoparticle nanocomposite with anti-EpCAM antibody.	Protothermal contrasting and visual readout	Breast cancer	[107]
AFP, CEA, CA125, and CA153.	Horse radish peroxidase (HRP)- O phenylene diamine H_2_O_2_	Electrochemical Immunodevice	Multiple	[119]
PSA	Bipolar electrode	electrochemiluminescence	Prostate cancer	[133]
microRNA-141 (miR-141) and microRNA-21 (miR-21)	Metal–organic framework (MOF) conjugated bio-probe, methylene blue (MB) and ferrocene (Fc) with distinguishable electrochemical signal,	Elctrochemical	Early detection of cancer	[134]
CEA	NH2-G/Thi/AuNPs nanocomposites modified electrode	Electrochemical	Multiple	[114]
miRNA-21	Positively charged conjugated polyelectrolyte (CPEs) “poly(3-alkoxy-4-methylthiophene)” (PT)	Colometric Through Naked Eye	Lung Cancer	[121]
NMP22 and BTA	Antibodies	Colometric With Naked Eye	Bladder Cancer	[60]
miRNA-21	DNA-templated Ag/Pt nanoclusters (DNA-Ag/Pt NCs),	Colometric Through Naked Eye	Lung Cancer	[135]
miRNA-21 and miRNA-31	Duplex-specific nuclease (DSN)	Laser-induced fluorescence (LIF)	miRNAs in cancer cells	[136]
blood cancer cells and skin cancer cell	photonic crystal fiber (PCF)	optical	blood and skin	[137]
Neuron-specific enolase (NSE)	NH2-G/Thi/AuNPs nanocomposites modified electrode	electrochemical detector and Android’s smartphone	Lung Cancer	[115]
cancer antigen 125 (CA125)	reduced graphene oxide/thionine/gold nanoparticles (rGO/Thi/AuNPs) nanocomposites coated working electrode	electrochemical	ovarian cancer, lung cancer, endometrial cancer and breast cancer	[118]
CEA	plasma separation	optical:raman scattering readout	Multiple	[120]
free hydrogen sulfide in prostate cancer cells	polyvinylpyrrolidone (PVP) membrane containing silver/Nafion	Colorimetric	Prostate cancer	[138]
PSA	multi wall carbon nanotubes MWCNTs activated PSA antibody (monoclonal antibody of the prostate specific antigen)	Electrochemical: Bench top multimeter	Prostate cancer	[102]
CEA	Anti CEA	Colorimetric	Multiple	[139]
PEAK1	Anti PEAK1	Colorimetric using gold nps	pancreatic cancer	[106]
PEAK1	nanomaterial graphene oxide coated electrode immobilized with anti-PEAK1	Electrochemical	pancreatic cancer	[140]
CEA PSA	[Ru(bpy)3]2+-labeled signal antibody CEA and PSA	Electrochemiluminescence	Multiple	[141]
Cytochrome c (Cyt c)	Cyt c aptamer and Raman reporter Cy5-labeled complementary DNA	optical:raman scattering	Lung Cancer	[142]
CEA and NSE	DNA aptamer	Electrochemical	Multiple	[116]
EGFR	anti-EGFR aptamers	Electrochemical	gastric, breast, ovarian, and colorectal cancers	[87]
MCF-7 cells	Aptamer-modified electrode	Electrochemiluminescence	Breast cancer	[143]
VEGF-C	NMB/NH2-SWCNT/AuNps modified Working electrode	Electrochemical	Cancer progression	[144]
urokinase plasminogen activator	graphene-AuNP platform and fluorescence of quantum dots	Colorimetric	Cancer progression	[145]
Micro RNA MiR-17	“light-switch” molecule [Ru(phen)2dppz]2+ modified electrode.	Electrochemiluminescence	Breast cancer	[146]
Osteopontin	Biotinylated aptamer for precapture and antibody for detection	Optical through naked eye	Cancer prognosis	[147]
Diphenylthiocarbazone	CuO NPs-labeled seconday Anibodies captured by antibodies	Fluorescence resonance energy transfer (FRET)	Prostate cancer	[148]
AFP and MUC16	AuNP labeling and anti-AFP and anti-MUC16 antibodies	colorimetric spot test	Multiple types	[149]
Perilipin-2	Gold nanorattles with PLIN-2 assay	Plasmonic biosensor	Renal cancer	[144]
CA 125	Ag/rGO nano-ink based electrodes with anti-CA	Electrochemical	Ovarian Cancer	[150]
CEA	Graphene-PEDOT:PSS modified electrode	Electrochemical	Multiple	[151]

## Data Availability

Not applicable.

## Abbreviations

The following abbreviations are used in this manuscript:

PoCT	Point of Care Testing
CEA	Carcino Embryonic Antigen
MCF-7	Michigan Cancer Foundation
AFP	Alpha-fetoprotein
MWCNT	Multi-walled carbon nano tubes
CA125	Cancer antigen 125
CA153	Carbohydrate antigen 153
PSA	Prostate-specific antigen
HRP	Horse radish peroxidase
MOF	Metal–Organic Framework
miR-141	microRNA-141
miR-21	microRNA-21
NSE	Neuron-specific enolase
PEAK1	Pseudopodium-enriched atypical kinase one
EGFR	Epidermal Growth Factor Receptor
Cyt C	Cytochrome c
PCF	Photonic crystal fiber
DSN	Duplex-specific nuclease
CPE	Conjugated polyelectrolyte

VEGF-C	Vascular endothelial growth factor C
NMB	New methylene blue
NH2-SWCNTs	Amino-functional single-walled carbon nanotubes
AuNPs	Gold nanoparticles
